# A two-stage deep learning architecture for radiographic staging of periodontal bone loss

**DOI:** 10.1186/s12903-022-02119-z

**Published:** 2022-04-01

**Authors:** Linhong Jiang, Daqian Chen, Zheng Cao, Fuli Wu, Haihua Zhu, Fudong Zhu

**Affiliations:** 1 Stomatology Hospital, School of Stomatology, Zhejiang University School of Medicine, Zhejiang Provincial Clinical Research Center for Oral Diseases, Key Laboratory of Oral Biomedical Research of Zhejiang Province, Cancer Center of Zhejiang University, Hangzhou, 310006 China; 2 School of Computer Science and Technology, Zhejiang University of Technology, Hangzhou, 310006 China; 3 College of Computer Science and Technology, Zhejiang University, Hangzhou, 310006 China

**Keywords:** Periodontitis, Alveolar bone loss, Deep learning, Convolutional neural network

## Abstract

**Background:**

Radiographic periodontal bone loss is one of the most important basis for periodontitis staging, with problems such as limited accuracy, inconsistency, and low efficiency in imaging diagnosis. Deep learning network may be a solution to improve the accuracy and efficiency of periodontitis imaging staging diagnosis. This study aims to establish a comprehensive and accurate radiological staging model of periodontal alveolar bone loss based on panoramic images.

**Methods:**

A total of 640 panoramic images were included, and 3 experienced periodontal physicians marked the key points needed to calculate the degree of periodontal alveolar bone loss and the specific location and shape of the alveolar bone loss. A two-stage deep learning architecture based on UNet and YOLO-v4 was proposed to localize the tooth and key points, so that the percentage of periodontal alveolar bone loss was accurately calculated and periodontitis was staged. The ability of the model to recognize these features was evaluated and compared with that of general dental practitioners.

**Results:**

The overall classification accuracy of the model was 0.77, and the performance of the model varied for different tooth positions and categories; model classification was generally more accurate than that of general practitioners.

**Conclusions:**

It is feasible to establish deep learning model for assessment and staging radiographic periodontal alveolar bone loss using two-stage architecture based on UNet and YOLO-v4.

## Background

Periodontal disease is among the most prevalent diseases of humankind globally; it affects billions of individuals and has heavy health and economic burdens. Periodontitis is the main cause of missing teeth in adults [1, 2], and most intraoral teeth may be affected with disease progression. Furthermore, as a chronic infectious disease, periodontitis is the sixth most common type of inflammatory disease [3] and is a risk factor or indicator for various systemic diseases, such as cardiovascular disease [4], diabetes mellitus [5], respiratory system infection [6], and digestive disease [7].

In the early stage, the symptoms of periodontal disease are not obvious and are sometimes ignored or missed, leading to the continued and irreversible development of the disease as it remains untreated, resulting in tooth mobility, loss, or even systemic disease. Timely and appropriate treatment based on early diagnosis and correct staging is critical for the control of periodontal disease.

The diagnosis and staging classification of periodontitis are mainly based on the state of periodontal alveolar bone resorption, including the level, shape, and location [8], which can be performed clinically with a periodontal probe. Since alveolar bone loss is often hidden behind the periodontal tissue and inaccessible, X-ray radiography, as a common aid applied to detect and assess the bone loss that is irreplaceable [9].

The bitewings and periapical X-rays focus on the details of the mouth area, such as one or several teeth, while the panoramic X-rays screen the whole dentition, jaws and bone structure with faster shooting and less radiation exposure. Therefore, the panoramic films are currently regarded as the most common and important radiology method in clinical dental evaluation, and have huge potential advantages in whole oral dental disease screening. It has been demonstrated that the intraoral and panoramic radiographic periodontal bone loss (PBL) results are largely in agreement with each other [10].

However, for various reasons (filming angle, structural overlap, physician ability, personal subjectivity, etc.), PBL detection on radiographs is marred by the limited accuracy of individual examiners and the low reliability between different examiners [11], especially general dentists, as demonstrated by a large range of studies and by various reference tests [12]. Therefore, a diagnosis system is needed to evaluate dental image data. This allows a reliable and accurate assessment of PBL on dental X-rays. Considering the large amount of human and economic resources required for a systematic, comprehensive, consistent and reliable assessment, the automatic assisted diagnosis system seems to play an important role.

In the past decade, with the advancement of artificial intelligence (AI) information technology and its integration with medicine, research on AI-assisted medical diagnosis models based on deep learning networks has shown potential for widespread applications.

Recent advances in deep learning models based on convolutional neural networks (CNNs) have shown potential for use in the automated identification and quantification of radiologic and pathologic features to improve diagnostic consistency and standardization of care. CNNs also have the potential to provide quantifiable outcomes, for example, to detect pulmonary nodules on CT imaging [13], hepatocellular carcinoma on multiphasic contrast-enhanced MRI [14], skin lesions in clinical skin screenings [15], or coronavirus disease 2019 indications in computed tomography images [16].

In dentistry, CNNs have been employed in the detection of caries in periapical X-rays and panoramic X-rays, as well as apical lesions and PBL on periapical X-rays, all with acceptable to high accuracy [17, 18]. To date, there have been limited attempts at automated assessments of PBL in dental radiographs by using deep learning; also, previous studies were committed to detection or trisection classification of alveolar bone height loss [17, 19–22]. Due to the inconsistency with the new staging framework widely accepted and used in clinical practice, the significance of these models for clinical diagnosis and decision-making is limited.

On the other hand, the shape (vertical type) and position (furcation lesions) of alveolar bone resorption have not been taken into consideration in previous studies. Both the shape and position are essential for the correct staging of periodontitis and appropriate clinical treatment. Vertical absorption and furcation lesions indicate possible local promoting factors, such as abnormal anatomy or occlusal interference, which require careful examination and corresponding interventions to address the risk factors [23, 24].

Therefore, we conducted this research to explore an automatic, comprehensive and correct radiographic bone loss staging system. In summary, the current study applied UNet to automatically identify and segment the tooth position on the panoramic film to reduce the interference of adjacent structures in the recognition process; used YOLO-v4 to automatically identify key points of each tooth (the cementoenamel junction (CEJ), apical point, and alveolar crest) to accurately calculate the degree of alveolar bone height reduction; used YOLO-v4 to automatically detect the shape of alveolar bone resorption (vertical type) and bone resorption at the furcation (furcation lesions); and finally aimed to comprehensively and accurately assess radiological PBL. The main contributions of this work are threefold. (1) We were the first to seek an automatic diagnosis system for PBL with special shapes and positions (vertical and furcation lesions). (2) We adopted the widely accepted stage classification standard advocated by the American Academy of Periodontology and the European Federation of Periodontology in 2017, with greater significance in guiding clinical practice. (3) We correctly calculated the percentage of radiographic bone loss after detecting the key points of each tooth in panoramic films so that the condition could be accurately staged.

## Methods

### Data set

Panoramic radiographs of each patient were acquired in 2018 using a dental panoramic X-ray machine (Orthopantomograph OP 100D, Instumentarium Corporation, Tuusula, Finland) at the Affiliated Stomatology Hospital, Zhejiang University School of Medicine. We prepared a total of 640 panoramic radiographs excluding the images of patients with primary or mixed dentition. The panoramic radiographs were collected retrospectively after identifiable patient information was removed. The study was approved by the Medical Ethics Committee of the Affiliated Hospital of Stomatology, School of Medicine, Zhejiang University (ChiCTR2100044897) and was conducted in compliance with the ICH-GCP principles and the Declaration of Helsinki (2013). The data collection and all experiments were performed in accordance with the relevant guidelines and regulations.

The images were randomly separated into a training set (80%) and a test set (20%) before data augmentation. The training set was used for CNN training of detection, and the testing set was used to evaluate the final trained model.

### Periodontist reading and labelling

Our staging of reduced alveolar bone height was based on the maximum PBL detectable on x-ray, expressed as a percentage of root length.

Each radiograph was read by three periodontists, each with more than 3 years of clinical experience, and 6 points were manually determined for each tooth to calculate the percentage of PBL and stage alveolar bone reduction. These points were the mesial and distal CEJ, root apex, and the deepest alveolar crest, respectively (for unirooted teeth, the mesial and distal root apex overlap was in the same position), as shown in Table 1.

**Table 1:** The meaning of key point abbreviations

Point	Reference
d1	Distal CEJ
d2	Distal alveolar crest
d3	Distal root apex
m1	Mesial CEJ
m2	Mesial alveolar crest
m3	Mesial root apex

The final label was determined based on consensus between the periodontists, i.e., different opinions on the point position were resolved by periodontists’ repeating their evaluation, and then all of the labels were reviewed and revised (addition, deletion, and confirmation) by a fourth periodontist. The examiners were instructed in person and calibrated using a handbook (describing how to use the annotation tool and how to annotate caries lesions, as well as how to discriminate these lesions from other entities) before they performed labelling and annotating tasks. Misplaced, overlapping teeth in the dataset were also included after careful identification and labelling.

**Fig. 1:**
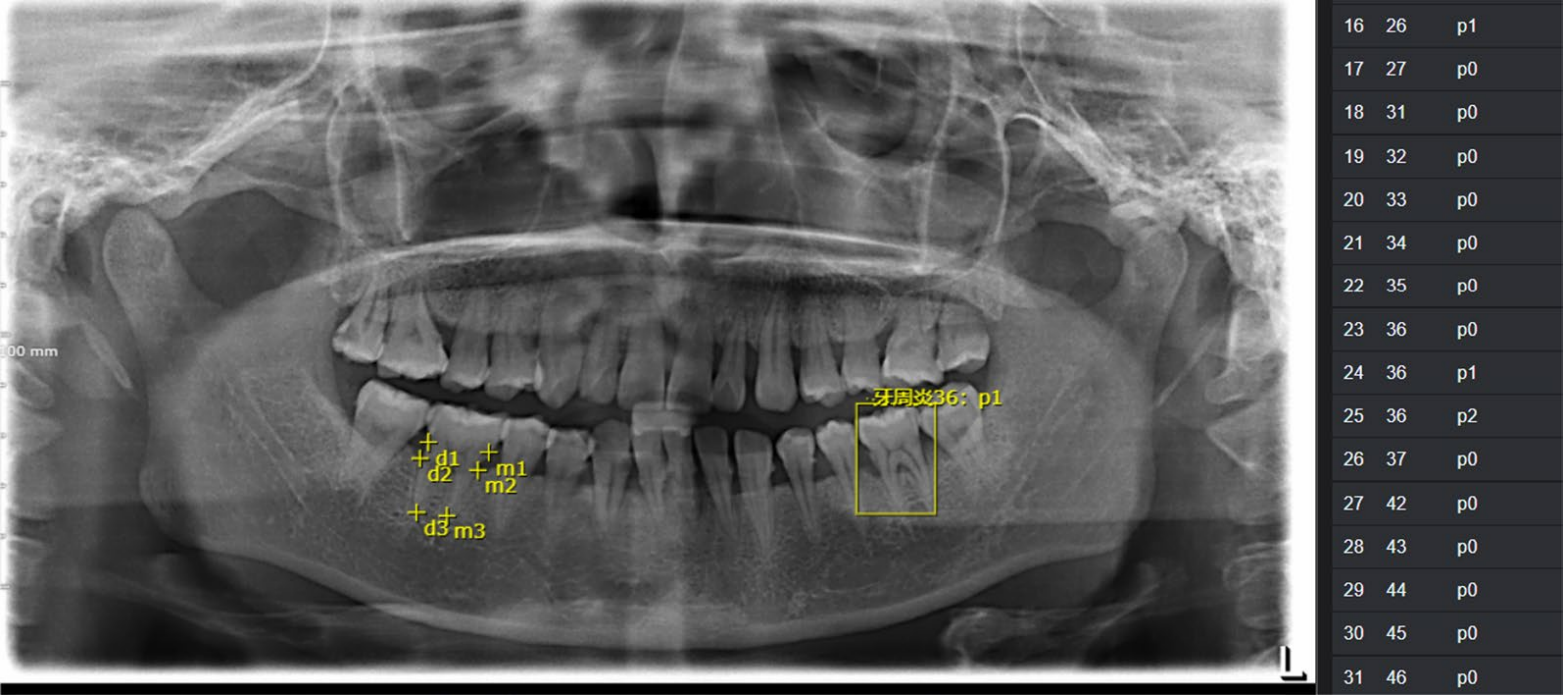
Labels on the key points of each tooth and a tooth with vertical alveolar reduction or furcation lesions on the panoramic radiographs (mandibular first molars were taken as examples) are shown

Additionally, examiners framed and labelled teeth with vertical alveolar reductions and furcation lesions (Fig. 1).

### Standard of staging periodontitis

The staging standard of the degree of alveolar bone resorption is based on six key points: m1, m2, m3, d1, d2, and d3 (for the mesial and distal CEJ, alveolar crest and root apex; Table 1). The six points were divided into 2 groups, d1-d2-d3 and m1-m2-m3, to calculate the mesial and distal PBL% (Table 2). For every tooth, two PBL% values (one for the mesial and one for the distal) were determined, and the larger PBL% was adopted as the basis for staging [21]. According to the Consensus of the Classification of Periodontal and Peri-Implant Diseases and Conditions [9], PBL% led to the stage result (Table 2).

**Table 2:** Calculation formula of PBL% and classification criteria

PBL%*	Stage
< 15%	I
15–33%	II
> 33%	III/IV

*PBL% = MAX (m1 − m2/m1 − m3, d1 − d2/d1 − d3)

When the distance between the alveolar bone and CEJ was within 2 mm, the patient was not clinically diagnosed as having alveolar bone resorption. However, considering the different shooting angles and zoom ratios of panoramic films, the absolute value of 2 mm is difficult to accurately define on the panoramic film. Therefore, this study did not distinguish between non-absorption and Stage I absorption.

### Model training

#### Data augmentation

Before model training, we performed data augmentation to make the model more accurate by modifying the images. The images were flipped horizontally and vertically and rotated. Therefore, the amount of data for deep learning was increased to 4 times that of the original amount.

#### Tooth segmentation

In view of the excessive interference information in the panoramic film, the first stage is the automatic detection and segmentation of tooth to reduce the interference of other structures. The first step is to identify every tooth contour in a panoramic film and cut into segments with single tooth automatically using the UNet network, which can combine deep and shallow information. Deep methods can provide the contextual semantic information of the segmentation object in the entire image and reflect the characteristics of the relationship between the object and its environment. In addition, medical images provide relatively little data, and the underlying features are more important. Pertinently, shallow information can provide more meticulous features for segmentation, such as gradients. After identifying the contours of the teeth, teeth fragments are isolated by expanding 20 pixels in all directions along the most prominent point of the contour of each tooth.

#### Object detection

The second stage is object detection.

The first part involves the use of CSPDarkNet, which can extract rich feature information from the input image. Notably, the interior of the network improved the information flow of dense blocks and transition layers, thus enhancing the learning capacity of the network, optimizing back propagation, and improving processing speed and memory.

The second part entails the use of the spatial pyramid pooling module + path aggregation network (SPP + PAN), which is can fuse feature information of different scales. SPP can enhance the model's detection of objects of different scales so that objects of different sizes and scales can be identified. The PAN proposes a two-way integration method that integrates bottom-up and top-down methods.

The third part involves the use of YOLO Head, which is employed for the final inspection. This part generates the final output vector with class probabilities, object scores and bounding boxes.

#### Calculation and staging

**Fig. 2:**
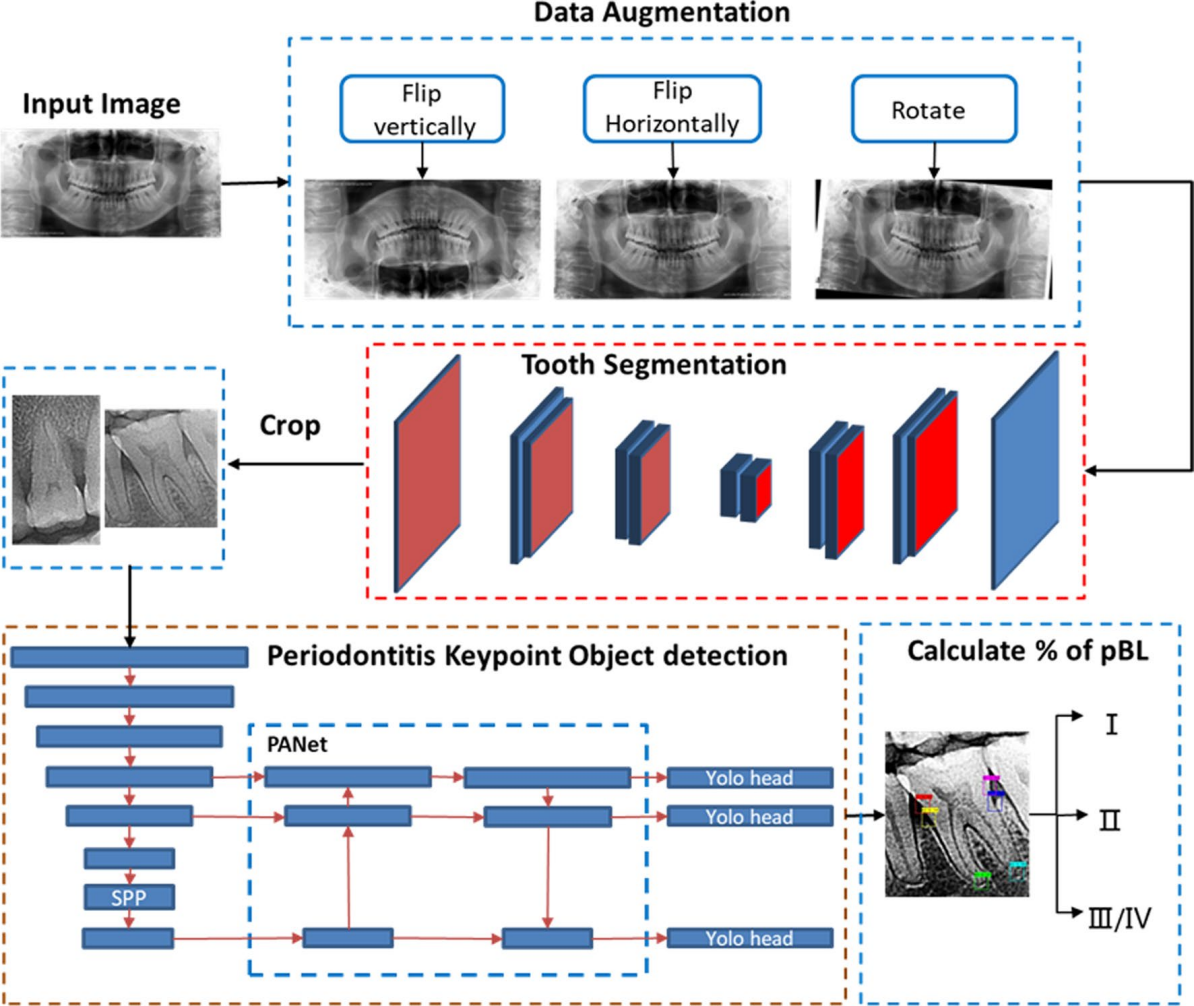
Workflow of the model training

Then followed by calculation of the percentage of periodontal alveolar bone loss and the staging of periodontitis. Based on the 6 key points detected for each tooth, the PBL% was mathematically calculated according to the aforementioned formula (Table 2) and divided into the corresponding categories (Fig. 2).

### Comparison with dentists

A cohort of three general dentists, all working in the Affiliated Stomatology Hospital, Zhejiang University School of Medicine for at least 3 years, was used as a comparator group to so that the relative performance of the neural network could be compared against that of individual dentists. Each of the participants independently classified PBL staging. It is worth noting that staging by both the model and dentists was determined only based on the severity, radiographic bone resorption.

### Metrics and statistical analysis

The diagnostic performance of the model and dentists was compared to the periodontists’ findings using confusion matrices. We calculated accuracy, precision, sensitivity, specificity, F1, and average precision (AP) and compared and analysed the diagnostic metrics between the model and three dentists using the chi-square test. Additionally, we evaluated the consistency of the three dentists’ diagnoses using the intraclass correlation coefficients (ICCs). Statistical analyses were performed with SPSS 24.0. Statistical significance level was set at *p* < 0.05.

## Results

**Fig. 3:**
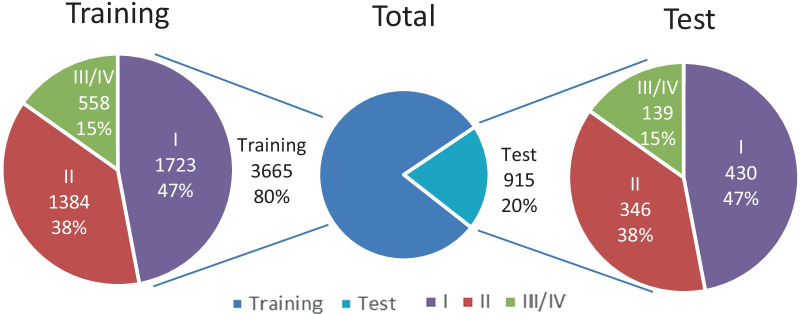
Whole distributions of data

**Fig. 4:**
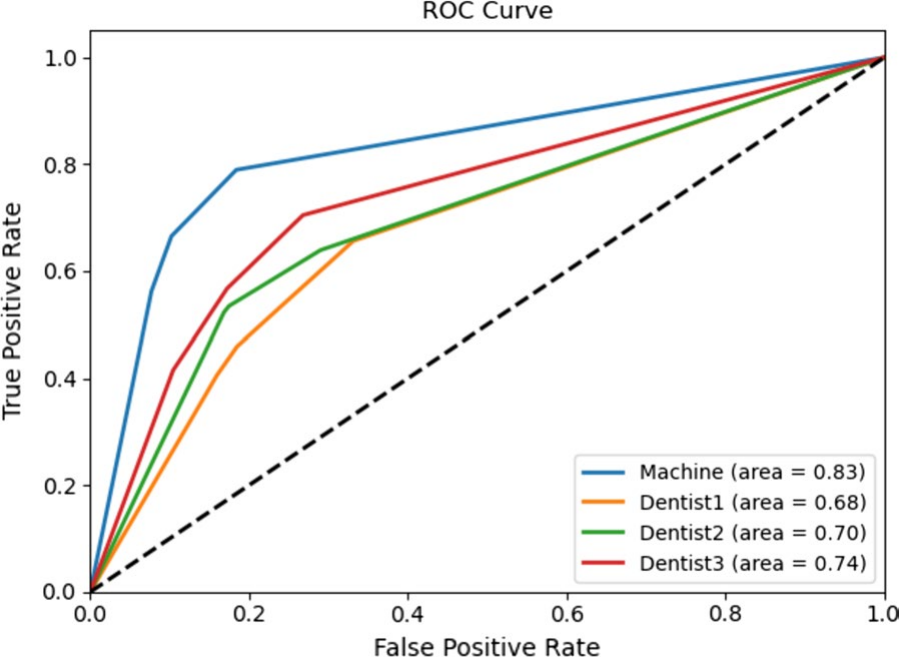
Receiver operating characteristic (ROC) curves for the model and dentists. The model was evaluated against the reference test with respect to sensitivity and specificity. The classification ability was further summarized by the area under curve (AUC) at the bottom right

We evaluated the performance of PBL% classification and vertical and furcation lesions recognition respectively. Table 3 and Fig. 3 show the distribution of periodontal lesions and their classifications in the reference dataset. Table 4 shows the performances of the model on PBL% stage classification in different teeth. Table 5 and Fig. 4 analyses the performances of the model and general dentists in PBL% stage classification in the test set. Table 6 summarizes separately metrics results of YOLO-v4 in vertical resorption and furcation lesion detection.

**Table 3:** Data set (segmentations)

	Training	Test	Total
I	1723	430	2153
II	1384	346	1730
III/IV	558	139	697
Vertical	497	125	622
furcation	544	137	681

**Table 4:** Performance of the model at different tooth positions

	Accuracy	Precision	Sensitivity	Specificity	F 1
Total	0.77	0.77	0.77	0.88	0.77
Maxillary anterior	0.81	0.80	0.81	0.89	0.80
Maxillary premolar	0.78	0.80	0.77	0.88	0.78
Maxillary molar	0.71	0.72	0.71	0.85	0.71
Mandibular anterior	0.71	0.72	0.72	0.85	0.72
Mandibular premolar	0.79	0.80	0.82	0.89	0.81
Mandibular molar	0.78	0.78	0.78	0.88	0.78

**Table 5:** Comparison between the model and dentists in different stages

	The model				Dentists’ mean (min–max)			
	I	II	III/IV	Total	I	II	III/IV	Total
Accuracy	0.88	0.79	0.87	0.77	0.78 (0.75–0.82)	0.64 (0.60–0.67)	0.76 (0.73–0.79)	0.59 (0.54–0.64)
Precision	0.77	0.78	0.76	0.77	0.55 (0.49–0.65)	0.66 (0.62–0.72)	0.57 (0.52–0.63)	0.6 (0.55–0.65)
Sensitivity	0.76	0.75	0.81	0.77	0.57 (0.54–0.59)	0.46 (0.33–0.52)	0.82 (0.69–0.88)	0.6 (0.54–0.66)
Specificity	0.92	0.82	0.90	0.88	0.85 (0.82–0.90)	0.79 (0.71–0.84)	0.74 (0.67–0.83)	0.79 (0.77–0.82)
F 1	0.77	0.77	0.78	0.77	0.56 (0.51–0.62)	0.53 (0.43–0.6)	0.66 (0.65–0.69)	0.59 (0.53–0.64)

**Table 6:** Performance of YOLO-v4 in vertical PBL and furcation PBL detection

	Precision	Sensitivity	F1	AP
Vertical PBL	0.88	0.51	0.64	0.52
Furcation PBL	0.94	0.75	0.83	0.74

First, the PBL% stage classification results for the model in different positions was presented. As shown in Table 4, the performance of the model was entirely acceptable with an overall accuracy of 0.77, and differed in different teeth. In maxillary anterior, premolar and mandibular posterior teeth, the accuracy of the model was relatively high at 0.78–0.81, and in maxillary molars and mandibular anterior teeth, the accuracy was lower at 0.71. The same results were found for the precision, sensitivity, specificity, and F1-score metrics.

Second, we compared the PBL% staging performance of the model and dentists. Overall, there was little difference in specificity, but the model obtained better accuracy, precision, sensitivity and F1 scores than the dentists, especially in stage I and II lesions, while the results showed significant difference (CI:95%, *p* < 0.05) (Table 5). In stage I lesions, the sensitivity of the model was 0.76, while it was 0.57 for dentists. For stage II lesions, the sensitivity of the model was 0.75, while it was 0.46 for dentists. For stage III lesions, the sensitivity of the model was 0.81, while it was slightly higher at 0.82 for dentists. It seemed that the model seemed was more sensitive in detecting stage I and II lesions, with little advantage in stage III lesions. The same results were found for the accuracy metrics. The Fig. 4 shows the receiver operating characteristic (ROC) curves for the model and the dentists, which allows to graphically compare the classification ability of the machine model and the dentists.

Finally, we evaluated the metrics of vertical resorption and furcation lesion detection (Table 6). The precision in furcation PBL was 0.94 and sensitivity was 0.75, which was considered satisfactory. For the vertical type, the precision and specificity of YOLO-v4 model were 0.88 and 0.51, respectively.

## Discussion

In 2017, the American Academy of Periodontology and the European Federation of Periodontology proposed a new definition and classification framework for periodontitis based on a multidimensional staging and grading system [8]. This widely accepted consensus proposed that an individual case of periodontitis should be further characterized using a matrix that describes the *stage* and *grade* of the disease.

*Stage* assesses two dimensionsof periodontitis: severity and complexity. The severity score is primarily based on clinical attachment loss and bone loss. The complexity score is based on the local treatment complexity, factors as vertical defect and furcation involvement are taken into count. Currently, staging is largely determined by the loss of periodontal tissue, which means the severity of the disease at the time. Staging is the basis for formulating patient treatment plans and interval between periodontal supportive treatment visits based on scientific evidence [25]. In the early stage, basic treatment works well. With periodontal disease progression, the treatment plan becomes more complicated, and the prognosis worsens. Thus, comprehensive screening for early diagnosis and precise staging for appropriate treatment are also important for the control of periodontal disease.

The *grade*, however, provides supplemental information on the patient's risk factors and rate of progression. It is determined based on primary criteria represented by direct or indirect evidence of periodontitis progression. Direct evidence is based on longitudinal data (including radiographic bone loss or clinical attachment loss) available, in many cases, in the form of older diagnostic quality radiographs. Indirect evidence is based on the assessment of bone loss at the worst affected tooth in the dentition as a function of age (measured as radiographic bone loss in percentage of root length divided by the age of the subject). To a certain content, radiographic bone loss is also basic for description of aggressiveness of the disease.

Therefore, we trained an AI model that could intelligently identify the key points for judging the percentage of periodontal bone resorption and then calculate the accurate percentage according to the formula to accurately stage periodontitis. On the other hand, the AI model could output reading results stably based on the same standard when facing a large number of films, with excellent potential for periodontitis screening.

In this study, UNet and YOLO-v4 were used to train a deep learning model for comprehensively diagnosing and accurately staging periodontal alveolar bone loss on panoramic oral films and compared with the diagnosis determined by dentists.

UNet is often used to evaluate biomedical images and performs well in medical image segmentation. YOLO-v4 used and combined some features, including weighted residual connections, cross-stage partial connections, cross mini-batch normalization, self-adversarial training and misactivation, mosaic data augmentation, DropBlock regularization, and CIoU loss, to improve CNN accuracy. Therefore, it could provide an efficient and powerful object detection model [26].

As shown in the previous section, the staging model generally performs well, as all metrics were satisfactory. The specificity is particularly superior; that is, the teeth predicted to be negative by the model were highly likely to be truly diagnosed as negative. This means that the model had a very small probability of missed detection, showing good screening potential. The model had different performance outcomes in different tooth positions, which may be related to the stretching and deformation of the image and the overlap of other local or adjacent structures in maxillary molars and mandibular anterior teeth. On average, the performance of the staging model was better than that of the dentists in addition to being stable, although the diagnosis results of the dentists were relatively consistent. The possible reason for this outcome was that the model accurately calculated the percentage based on the identified key points, while the physician estimated the percentage based on visual observation (simulating clinical scenarios). There was a difference in accuracy between the two, especially near the staging threshold. In different categories, the accuracy was different due to the difference in the height of the alveolar bone loss. Hence, models trained based on expert diagnostic criteria may perform better than ordinary general dentists.

The detection model of furcation lesions had acceptable results, while that of vertical absorption had relatively low specificity and high accuracy, which may have been related to the insignificant image characteristics of vertical absorption. Furcation lesions could be a strong reminder or warning of a missed diagnosis; that is, if the model predicted vertical absorption, there was a high probability of vertical absorption. If the dentists or radiologists re-examined or read the film carefully under the prompt of the positive result of the model, the vertical absorption that was missed previously was likely to be confirmed.

Compared with the published research, the advantages of this research are the following: we used new structures and models, combined with automatic tooth recognition and segmentation and key point object detection; this combination reduced the interference of irrelevant structures. We also calculated the percentage of PBL more accurately and achieved accurate staging. The classification standard was based on the consensus of the clinically widely recognized and widely used periodontitis staging framework, so that the research results were more consistent and relevant to the clinic. Also, the inspection content was more comprehensive. The study included the detection of PBL in specific parts and shapes related to diagnosis and decision-making.

However, this study still had many limitations. First, the research was not conducted in a real clinical environment. The data set was acquired retrospectively from radiological films. Also, these single-modal data model does not include clinical data necessary for a comprehensive and accurate diagnosis of periodontitis in clinical practice. Therefore, the model can only assist staging radiographic bone loss. Follow-up studies are needed to explore the fusion of radiological image data and clinical text data, to obtain a perfect diagnosis model. Second, the criteria were based on the results of professional and experienced periodontal specialists. The absence of a gold standard leads to possible diagnostic bias. In addition, there was no distinction between non-resorption of periodontal alveolar bone and stage I resorption in the radiological staging diagnosis because a distance of 2 mm could not be accurately measured on panoramic film. Furthermore, the model was not externally verified, which may lead to overfitting to the training data set, potentially resulting in an overestimation of the model’s performance [27, 28]. Before clinical application, a large number of external data sets are needed to optimize the model, and parameters should be adjusted according to different environment and equipment in medical institutions. Finally, the resulting model has not been used in the clinic.

Further work will focus on increasing the size of the data set, using three-dimensional images to improve model prediction accuracy, and combining clinical text information for further treatment decisions making and prognosis prediction.

## Conclusion

In summary, the well-trained deep learning architecture based on UNet and YOLO-v4 performed well in detecting and clarifying alveolar bone loss radiologically, and could assist dentists in comprehensive and accurate assessment for periodontal bone loss.

## Data Availability

The data are not publicly available due to privacy. The data presented in this study are available on request to the corresponding author.

## Abbreviations

PBL: Periodontal bone loss
AI: Artificial intelligence
CNN: Convolutional neural network
CEJ: Cementoenamel junction
SPP: Spatial pyramid pooling module
PAN: Path aggregation network
AP: Average precision
ICC: Intraclass correlation coefficients
ROC: Receiver operating characteristic
AUC: Area under curve

## References

[1] Lee J, Lee J, Choi J (2016). National dental policies and socio-demographic factors affecting changes in the incidence of periodontal treatments in Korean: a nationwide population-based retrospective cohort study from 2002–2013. BMC Oral Health.

[2] Lee J, Oh J, Choi J (2017). Trends in the incidence of tooth extraction due to periodontal disease: results of a 12-year longitudinal cohort study in South Korea. J Periodontal Implant Sci.

[3] Tonetti MS, Jepsen S, Jin L (2017). Impact of the global burden of periodontal diseases on health, nutrition and wellbeing of mankind: a call for global action. J Clin Periodontol.

[4] Chistiakov DA, Orekhov AN, Bobryshev YV (2016). Links between atherosclerotic and periodontal disease. Exp Mol Pathol.

[5] Taylor GW, Borgnakke WS (2008). Periodontal disease: associations with diabetes, glycemic control and complications. Oral Dis.

[6] Tan L, Tang X, Pan C (2019). Relationship among clinical periodontal, microbiologic parameters and lung function in participants with chronic obstructive pulmonary disease. J Periodontol.

[7] Hajishengallis G (2015). Periodontitis: from microbial immune subversion to systemic inflammation. Nat Rev Immunol.

[8] Tonetti MS, Greenwell H, Kornman KS (2018). Staging and grading of periodontitis: framework and proposal of a new classification and case definition. J Periodontol.

[9] Papapanou PN, Sanz M, Buduneli N (2018). Periodontitis: Consensus report of workgroup 2 of the 2017 world workshop on the classification of periodontal and peri-implant diseases and conditions. J Clin Periodontol.

[10] Persson RE, Tzannetou S, Feloutzis AG (2003). Comparison between panoramic and intra-oral radiographs for the assessment of alveolar bone levels in a periodontal maintenance population. J Clin Periodontol.

[11] Akesson L, Håkansson J, Rohlin M (1992). Comparison of panoramic and intraoral radiography and pocket probing for the measurement of the marginal bone level. J Clin Periodontol.

[12] Choi IGG, Cortes ARG, Arita ES (2018). Comparison of conventional imaging techniques and CBCT for periodontal evaluation: a systematic review. Imaging Sci Dent.

[13] Chae KJ, Jin GY, Ko SB (2020). Deep learning for the classification of small (≤2 cm) pulmonary nodules on CT imaging: a preliminary study. Acad Radiol.

[14] Bousabarah K, Letzen B, Tefera J (2021). Automated detection and delineation of hepatocellular carcinoma on multiphasic contrast-enhanced MRI using deep learning. Abdom Radiol (New York).

[15] Haenssle HA, Fink C, Toberer F (2019). Man against machine reloaded: performance of a market-approved convolutional neural network in classifying a broad spectrum of skin lesions in comparison with 96 dermatologists working under less artificial conditions. Ann Oncol Off J Eur Soc Med Oncol.

[16] Qayyum A, Razzak I, Tanveer M (2021). Depth-wise dense neural network for automatic COVID19 infection detection and diagnosis. Ann Oper Res.

[17] Khan HA, Haider MA, Ansari HA (2021). Automated feature detection in dental periapical radiographs by using deep learning. Oral Surg Oral Med Oral Pathol Oral Radiol.

[18] Lee J, Kim D, Jeong S (2018). Detection and diagnosis of dental caries using a deep learning-based convolutional neural network algorithm. J Dent.

[19] Chang H, Lee S, Yong T (2020). Deep learning hybrid method to automatically diagnose periodontal bone loss and stage periodontitis. Sci Rep.

[20] Kim J, Lee H, Song I (2019). DeNTNet: deep neural transfer network for the detection of periodontal bone loss using panoramic dental radiographs. Sci Rep.

[21] Krois J, Ekert T, Meinhold L (2019). Deep learning for the radiographic detection of periodontal bone loss. Sci Rep.

[22] Lee J, Kim D, Jeong S (2018). Diagnosis and prediction of periodontally compromised teeth using a deep learning-based convolutional neural network algorithm. J Periodontal Implant Sci.

[23] Nibali L, Sun C, Akcalı A (2018). The effect of horizontal and vertical furcation involvement on molar survival: a retrospective study. J Clin Periodontol.

[24] Rams TE, Listgarten MA, Slots J (2018). Radiographic alveolar bone morphology and progressive periodontitis. J Periodontol.

[25] Sanz M, Herrera D, Kebschull M (2020). Treatment of stage I-III periodontitis-The EFP S3 level clinical practice guideline. J Clin Periodontol.

[26] Bochkovskiy A, Wang C, Liao HM. YOLOv4: optimal speed and accuracy of object detection. 2020. https://arxiv.org/abs/2004.10934.

[27] England JR, Cheng PM (2019). Artificial intelligence for medical image analysis: a guide for authors and reviewers. AJR Am J Roentgenol.

[28] Park SH, Han K (2018). Methodologic guide for evaluating clinical performance and effect of artificial intelligence technology for medical diagnosis and prediction. Radiology.

